# Phosphatidylglycerol homeostasis in glycerol-phosphate auxotrophs of *Staphylococcus aureus*

**DOI:** 10.1186/1471-2180-13-260

**Published:** 2013-11-16

**Authors:** Joshua B Parsons, Jiangwei Yao, Pamela Jackson, Matthew Frank, Charles O Rock

**Affiliations:** 1 Department of Infectious Diseases, St. Jude Children’s Research Hospital, 262 Danny Thomas Place, Memphis TN 38105, USA

**Keywords:** Acyl carrier protein, PlsY, Acyl-phosphate, Phosphatidylglycerol, *S. aureus*

## Abstract

**Background:**

The balanced synthesis of membrane phospholipids, fatty acids and cell wall constituents is a vital facet of bacterial physiology, but there is little known about the biochemical control points that coordinate these activities in Gram-positive bacteria. In *Escherichia coli*, the glycerol-phosphate acyltransferase (PlsB) plays a key role in coordinating fatty acid and phospholipid synthesis, but pathogens like *Staphylococcus aureus* have a different acyltransferase (PlsY), and the headgroup of the major membrane phospholipid, phosphatidylglycerol (PtdGro), is used as a precursor for lipoteichoic acid synthesis.

**Results:**

The PlsY acyltransferase in *S. aureus* was switched off by depriving strain PDJ28 (Δ*gpsA*) of the required glycerol supplement. Removal of glycerol from the growth medium led to the rapid cessation of phospholipid synthesis. However, the continued utilization of the headgroup caused a reduction in PtdGro coupled with the accumulation of CDP-diacylglycerol and phosphatidic acid. PtdGro was further decreased by its stimulated conversion to cardiolipin. Although acyl-acyl carrier protein (ACP) and malonyl-CoA accumulated, fatty acid synthesis continued at a reduced level leading to the intracellular accumulation of unusually long-chain free fatty acids.

**Conclusions:**

The cessation of new phospholipid synthesis led to an imbalance in membrane compositional homeostasis. PtdGro biosynthesis was not coupled to headgroup turnover leading to the accumulation of pathway intermediates. The synthesis of cardiolipin significantly increased revealing a stress response to liberate glycerol-phosphate for PtdGro synthesis. Acyl-ACP accumulation correlated with a decrease in fatty acid synthesis; however, the coupling was not tight leading to the accumulation of intracellular fatty acids.

## Background

The maintenance of membrane lipid homeostasis is a vital process in bacterial metabolism [1]. The synthesis of membrane proteins and lipids is coordinated in *Escherichia coli* to ensure that the biophysical properties of the membrane remain constant regardless of the growth rate or environmental stress. Overexpression of the pathway enzymes does not increase the amount of lipid in the cell illustrating the stringent biochemical regulation of the pathway that precisely matches the rate of fatty acid synthesis to the demand for new membrane. The biochemical regulation of type II fatty acid synthesis (FASII) in bacteria is most completely studied in *Escherichia coli*[2-4]. The scheme that has emerged places the first committed step in membrane phospholipid synthesis, *sn*-glycerol-3-phosphate (glycerol-PO_4_) acyltransferase (PlsB), as a key regulatory point. How PlsB senses the requirement for new phospholipid is not completely understood, but one biochemical regulator is ppGpp [5], a global regulator of gene expression [6]. The consequences of regulation at the PlsB step are relayed to FASII by long-chain acyl-acyl carrier protein (ACP), a crucial allosteric regulator of two steps in initiation. The importance of acyl-ACP was first recognized by the expression of acyl-ACP thioesterases in *E. coli*, which leads to run-away FASII activity and the secretion of copious amounts of free fatty acids [7-9]. Long-chain acyl-ACPs act as potent feedback inhibitors of FASII by blocking the initiation of new acyl chains at the FabH step [10,11] and slowing the elongation of acyl chains by inhibiting acetyl-CoA carboxylase [12]. It is not clear whether this regulatory model for membrane lipid homeostasis in *E. coli* can be extended to Gram-positive bacteria. Notably, these organisms do not have a PlsB acyltransferase, but rather use a novel activated acyl donor, acyl-phosphate (acyl-PO_4_), produced by PlsX from the acyl-ACP end-products of FASII, and have a unique glycerol-PO_4_ acyltransferase, PlsY, which only uses acyl-PO_4_[13]. Precise control over fatty acid synthesis appears even more important for Gram-positive pathogens like *S. aureus*, because unlike *E. coli*, they lack a fatty acid catabolic pathway [14]. Expression of the genes responsible for phosphatidic acid biosynthesis in *Bacillus subtilis* and *S. aureus* is controlled by FapR [15], which releases from its DNA binding sites within the regulons multiple promoters when bound to malonyl-CoA [16,17]. Although the transcriptional regulation of lipid synthesis is understood in considerable detail, much less is known about the biochemical regulation of FASII or the coupling of fatty acid and phospholipid synthesis.

Glycerol-PO_4_ is the substrate for PlsY and a required precursor for membrane phospholipid synthesis, and is produced from dihydroxyacetone phosphate by glycerol-PO_4_ synthase (GpsA) [18]. Forty years ago Mindich [19] isolated a *S. aureus* glycerol auxotroph and demonstrated that phospholipid synthesis from [^14^C]acetate ceased abruptly following removal of the glycerol growth supplement, although free fatty acids continued to accumulate. Subsequent work revealed that the free fatty acids consisted primarily of 21-carbon branched-chain species that are longer than the normal 15–17 carbon fatty acids in normally growing cells [20]. Total protein synthesis continued following the removal of glycerol resulting in denser cells [20]. Protein synthesis also continued when glycerol was withheld from *Bacillus subtilis* glycerol auxotrophs illustrating that protein and lipid synthesis are not tightly coupled in Gram-positive bacteria [21,22]. A different approach was used by Paoletti et al. [23] who inactivated *plsY* in *B. subtilis* with an intact *plsY* gene under control of a regulated promoter. In this model, the inactivation of PlsY activity is not immediate or complete, but rather the strain must be grown for hours to deplete pre-existing PlsY protein. Nonetheless, fatty acid accumulation was detected in PlsY-depleted cells [23].

These earlier experiments did not investigate the effect of glycerol deprivation on either the membrane lipid composition or the level or composition of the lipid precursor pools. Because knowledge of these metabolic intermediates will provide insight into the role of PlsY in pathway regulation, we constructed a *gpsA* knockout in *S. aureus* to more precisely investigate the regulation of FASII and phospholipid metabolism in the absence of PlsY activity. The cessation of phospholipid synthesis does not blunt the continued metabolism of the principle membrane phospholipid, phosphatidylglycerol (PtdGro), resulting in a marked disruption of membrane phospholipid homeostasis. Long-chain acyl-acyl carrier protein (ACP) and malonyl-CoA accumulate following the block at PlsY, but fatty acid synthesis continues at a reduced rate reflected by the accumulation of intracellular fatty acids.

## Methods

### Bacterial strains and media

*S. aureus* strain RN4220 was obtained from Richard Novick [24]. Strain PDJ28 (Δ*gpsA*) was constructed as described previously [25]. A group II intron was inserted at bp 42 of the *gpsA* gene using the primer design software and plasmid system provided by Sigma-Aldrich (Targetron system) [26]. The presence of the insertions was verified by multiplex PCR using opposing primers located in the *gpsA* gene outside the intron insertion site and one primer inside the intron. The wild-type allele yields a product of 528 bp and the disrupting gene gives a product of 394 bp. RN minimal medium was used for broth cultures and consisted of M9 salts, 1 mM MgSO_4_, 10 mM CaCl_2_, 15 μM vitamin B_1_, 32 μM vitamin B_3_, 0.1% casein hydrolysate, 0.4% glucose, 0.1 mg/l biotin, 2 mg/l pantothenic acid, 10 μM FeCl_2_, 6 mg/l citrate, 10 mg/l MnCl_2_, 4 μg/l L-tryptophan, and 0.1 mg/l lipoic acid.

### Metabolic labeling

Phospholipids and fatty acids were labeled by the addition of 50 μCi [1-^14^C]acetate (50 Ci/mol) per 10 ml culture. For labeling of lipids before glycerol starvation, RN media supplemented with 0.1% glycerol and 50 μCi [1-^14^C]acetate (1 Ci/mol) per 10 ml culture was inoculated with strain PDJ28 to OD_600_ = 0.05 and grown to OD_600_ = 0.6. The cells were pelleted and washed with 50 ml RN media and used to inoculate cultures in RN media with and without 0.1% glycerol supplement for indicated time. For labeling of lipids after glycerol starvation, cells were washed as previously described, grown for 30 minutes before addition of 50 μCi [1-^14^C]acetate (50 Ci/mol) per 10 ml culture for indicated time. 10 ml samples were centrifuged, washed with 10 ml RN media and 10 ml H_2_O. Pellets were resuspended in 100 μl H_2_O and lipids extracted through the addition of 360 μl of chloroform:methanol:HCl (1/2/0.02) and incubated at room temperature for 20 minutes. 120 μl chloroform and 120 μl 2 M KCl were added to separate phases and after centrifugation, the organic phase was removed and radioactivity quantified by scintillation counting.

### Thin-layer chromatography

Radiolabelled lipids were analyzed by 1-dimensional and 2-dimensional thin-layer chromatography. The 1-dimensional system used to separate phospholipids from diacylglycerol and fatty acid was Silica Gel G layers developed with chloroform:methanol:acetic acid (98/2/1) and visualized using Bioscan imaging detector. The 2-dimensional system also employed Silica Gel G layers and was developed first with chloroform:methanol:water (65/25/4) and secondly tetrahydrofuran/dimethoxyethane/methanol/4 M ammonium hydroxide (10/6/4/1). The resulting thin-layer plate exposed to a PhosphoImager screen and visualized using a Typhoon 9200.

### Lipid mass spectrometry

Mass spectrometry of phospholipids was performed using a Finnigan TSQ Quantum (Thermo Electron, San Jose, CA) triple quadrupole mass spectrometer. Samples were prepared in 50:50 (v/v) chloroform:methanol. The instrument was operated in the negative ion mode. Ion source parameters were as follows: spray voltage of 3,000 V, capillary temperature of 270°C, capillary offset of 35 V, and the tube lens offset was set by infusion of polytyrosine tuning and calibration solution (Thermo Electron, San Jose, CA) in the electrospray mode. Acquisition parameters were as follows: full scan, scan range 600 – 100 *m*/*z*, scan time 0.5 s, peak width Q1 0.7 FWHM. Instrument control and data acquisition was performed with the Finnigan™ Xcalibur™ software (Thermo Electron, San Jose, CA).

### Mass spectrometry malonyl-CoA measurement

Cultures of strain PDJ28 were grown in RN medium supplemented with 0.1% glycerol to OD_600_ = 0.6. Cells were pelleted and washed with 50 ml RN medium to remove glycerol and used to inoculate RN medium with and without 0.1% glycerol. Cultures were grown for 120 minutes and harvested at room temperature. Cells were extracted using the Bligh and Dyer method [27], and 50 pmol of [^13^C_3_]malonyl-CoA (Stable Isotope Products; Isotec) was added. The aqueous phase was applied to a 100-mg 2-(2-pyridyl)ethyl functionalized silica gel column (Supelco) equilibrated with 2% acetic acid in methanol/water (1:1) [28]. The column was washed two times with 1 ml of equilibration buffer and 1 ml water. CoAs were eluted with 1 ml of 50% acetonitrile containing 15 mM ammonium hydroxide. Mass spectrometry of acyl-CoA was performed using a Finnigan TSQ Quantum (Thermo Electron) triple-quadrupole mass spectrometer [29]. The instrument was operated in positive mode using single ion monitoring (SIM) neutral loss scanning corresponding to the loss of the phosphoadenosine diphosphate from CoA species. The ion source parameters were as follows: spray voltage, 4,000 V; capillary temperature, 250°C; capillary offset, -35 V; sheath gas pressure, 10; auxiliary gas pressure, 5; and tube lens offset was set by infusion of the polytyrosine tuning and calibration in electrospray mode. Acquisition parameters were as follows: scan time, 0.5 s; collision energy, 30 V; peak width Q1 and Q3, 0.7 FWHM; Q2 CID gas, 0.5 mTorr; source CID, 10 V; neutral loss, 507.0 *m*/*z*; SIM mass of 855 *m*/*z* with a scan width of 10 *m*/*z* to capture the signals from both light and heavy malonyl-CoA, and SIM mass of 810 *m/z* with a scan width of 6 *m/z* to capture the signal of acetyl-CoA.

### ACP immunoblotting

Cultures of strain PDJ28 (Δ*gpsA*) and parent *S. aureus* strain RN4220 cells were grown to OD_600_ = 0.5 in RN minimum media with 1% glycerol supplementation at 37°C with rigorous shaking (225 rpm), and then split into 50 ml aliquots. Cells were washed twice with RN media. For PDJ28 without glycerol supplement and strain RN4220, cells were suspended in 50 ml of RN media. Strain PDJ28 was grown in RN media with 1% glycerol supplementation. Cells were grown for the indicated amount of time, pelleted, and resuspended in 125 μl of 25% sucrose and 50 mM Tris pH 7.0 on ice. Lysostaphin (25 μl of a 5 mg/ml) was added to the mixture, and incubated on ice for 15 minutes. Finally, the cells were lysed by adding 200 μl of 10% Triton X-100, 62.5 mM EDTA, and 50 mM Tris–HCl pH 7.5. The lysed cells were centrifuged at 40,000 g for 30 minutes. The supernatant, in native loading buffer, was loaded onto a 2.5 M urea, 15% acrylamide gel. The amount of supernatant loaded in each sample is adjusted to OD_600_ such that total protein is similar for each lane.

### Gas chromatography

Cultures of strain PDJ28 cells were grown in RN media with 1% glycerol supplement at 37°C with rigorous shaking (225 rpm). Cells were grown to OD_600_ of 0.5, aliquoted to 50 ml cultures, and washed twice with RN media. Then, one cell aliquot was grown in RN minimum media and another aliquot was grown in RN minimum media supplemented with 1% glycerol for an additional 2 hours. Cells were washed with phosphate-buffered slaine three times and harvested for lipids using the method of Bligh and Dyer [27]. The free fatty acids were separated from the other lipid species by thin-layer chromatography. Briefly, the lipid extract was loaded onto Silica Gel G plates (Analtech) and chromatographed in chloroform:methanol:acetic acid (98/2/1) solvent mixture. The silica gel at R_f_ of 0.7 or higher was scraped off the plate to collect the free fatty acid fractions. The scraped silica was added to 1 ml water, and extracted 3 times with 1 ml hexane. The hexane fractions were collected and evaporated to obtain the free fatty acid samples. Fatty acid methyl esters were generated from the collected fatty acids by bringing up the fatty acid samples in 5 ml of 2% anhydrous HCl in anhydrous methanol and incubating overnight. The reaction was evaporated under nitrogen and brought up in 1 ml of distilled water. The water phase was extracted 3 times with hexanes. The hexane fractions were pooled and evaporated over nitrogen. The fatty acid methyl esters were analyzed by a Hewlett-Packard model 5890 gas chromatograph equipped with a flame ionization detector, and separated on 30 m × 0.536 mm × 0.50 μm DB-225 capillary column. The injector was set at 250°C, and the detector was at 300°C. The temperature program was as followed: initial temp 70°C for 2 min, rate of 20°C/min for 5 min (final 170°C), rate of 2°C/min for 10 min (final 190°C), hold at 190°C for 5 min, rate of 2°C/min for 15 min (final 220°C), hold at 220°C for 5 min. The identity of fatty acid methyl esters were determined by comparing their retention times with identified fatty acid methyl ester standards (Sigma-Aldrich). The compositions were expressed as weight percentages.

## Results

### Growth characteristics of *S. aureus* strain PDJ28 (Δ*gpsA*)

**Figure 1:**
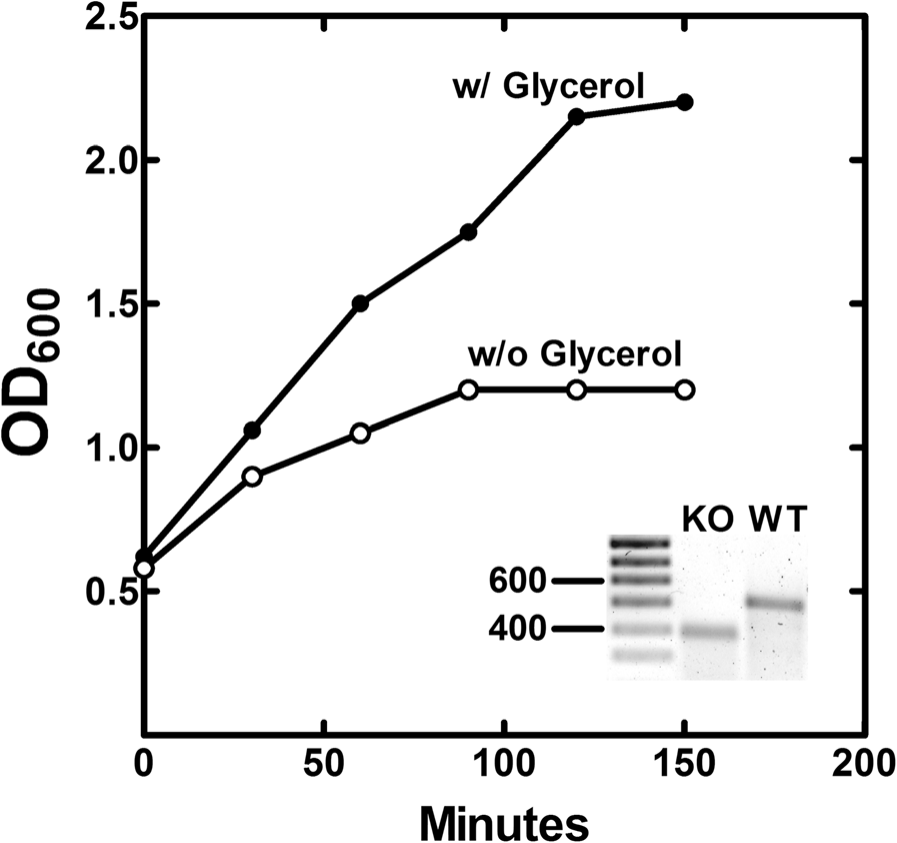
**Growth phenotype of the *gpsA *knockout strain.***S. aureus* strain PDJ28 (Δ*gpsA*) was grown in RN medium to an OD_600_ of 0.5 and the cells were harvested and washed to remove the glycerol supplement. The culture was split and resuspended in media either with or without 0.1% glycerol, and growth was followed as a function of time. The growth curve is representative example of the data obtained in duplicate experiments. The figure inset shows the multiplex PCR genotyping of the wild-type *gpsA* gene (528 bp) in strain RN4220 and the inactivated *gpsA* allele (394 bp) in strain PDJ28 as described under Methods.

The *S. aureus gpsA* gene (SA1306) was disrupted by the insertion of a Group II intron (see Methods). The insertion was confirmed by PCR genotyping showing the presence of the inactivating DNA insertion in the *gpsA* gene (Figure 1, *inset*). Strain PDJ28 was a glycerol or glycerol-PO_4_ auxotroph on agar plates (not shown). The growth of strain PDJ28 in RN media broth was followed after the removal of the glycerol supplement (Figure 1). The rate of cell growth immediately slowed, and then ceased after 90 min. These growth characteristics were similar to the growth phenotypes of the *gpsA* knockouts previously isolated in *E. coli*[30], *B. subtilis*[22] and *S. aureus*[20].

### Alterations in membrane phospholipid homeostasis following glycerol removal

**Figure 2:**
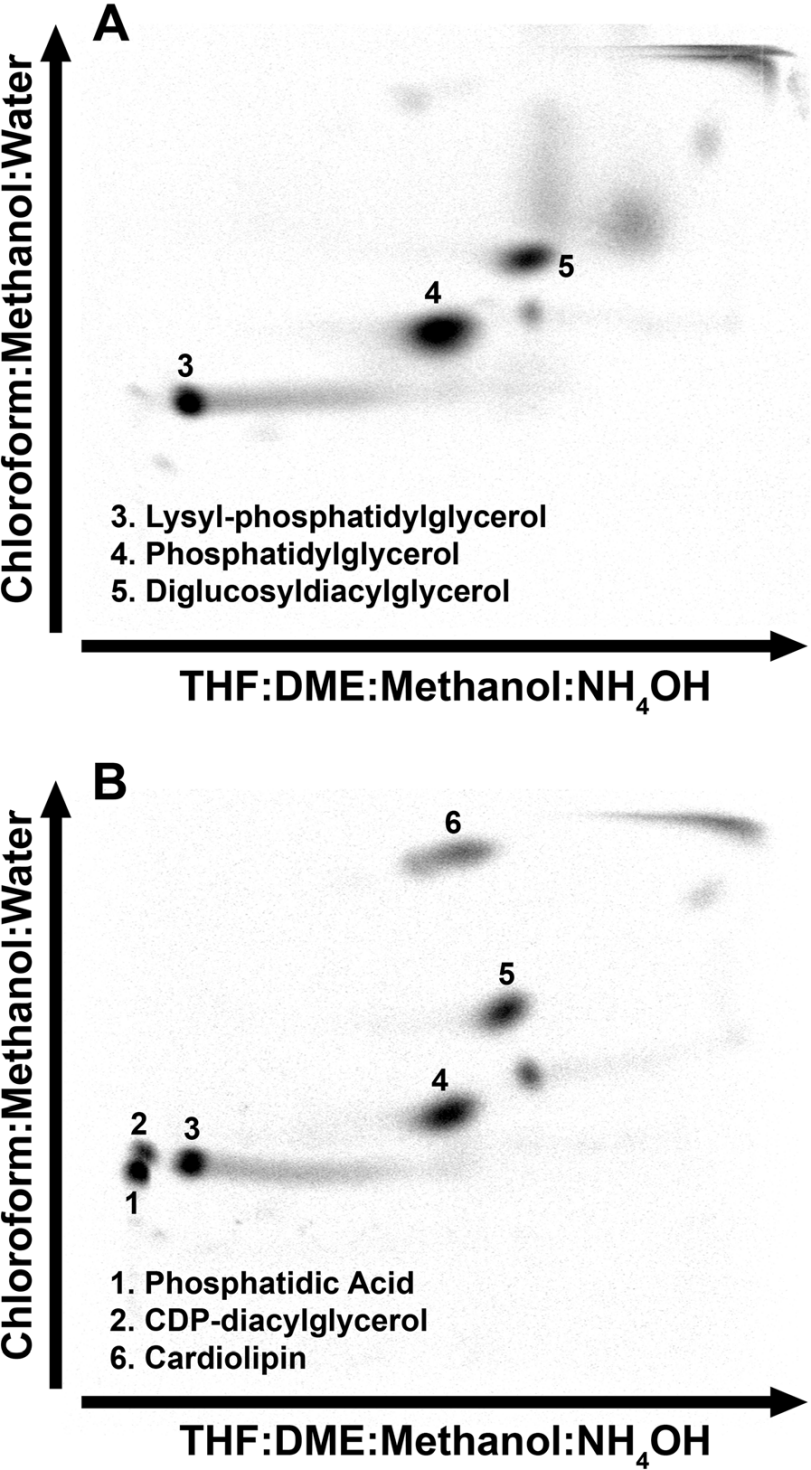
**Altered membrane lipid composition of strain PDJ28 following the removal of the glycerol supplement.** Strain PDJ28 (Δ*gpsA*) was labeled with [^14^C]acetate in the presence of glycerol to an OD_600_ of 0.6. The cells were then washed and resuspended in media either with **(A)** or without **(B)** the glycerol supplement, and after 180 min at 37°C, the cellular lipid composition was determined by 2-dimensional thin-layer chromatography of the extracted lipids. The distribution of radioactivity was determined using a PhosphoImager screen and a Typhoon 9200.

**Figure 3:**
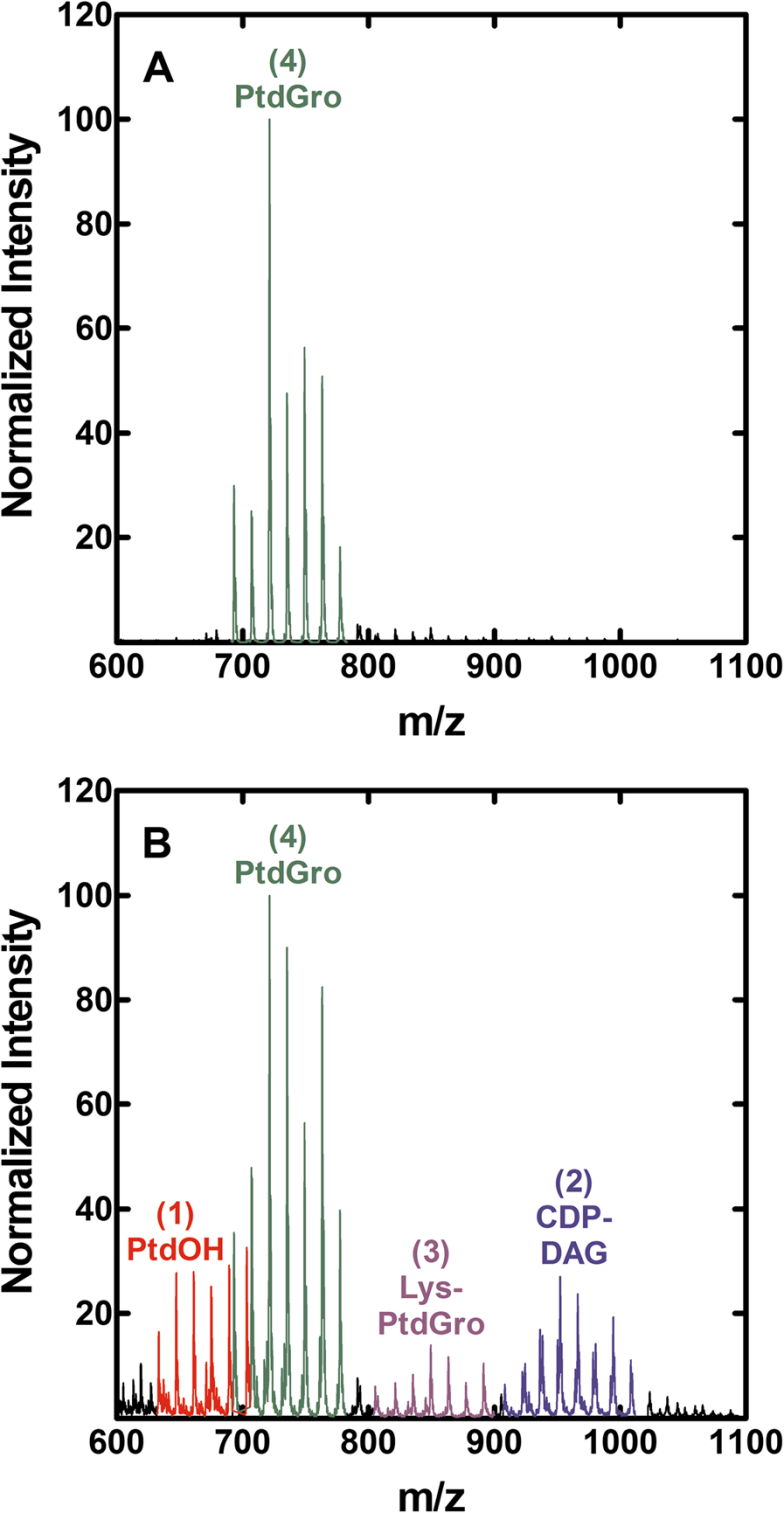
**Mass spectrometry identification of PtdOH and CDP-DAG accumulation following the removal of the glycerol supplement.** The identity of the two new polar phospholipid species that appeared in glycerol–starved cells was confirmed by mass spectrometry of the phospholipid fraction in the presence **(A)** or absence **(B)** of the glycerol supplement. Samples were prepared and analyzed by mass spectrometry as described in Methods.

The removal of the glycerol supplement from strain PDJ28 (Δ*gpsA*) had a significant impact on the membrane phospholipid composition. The metabolism of existing membrane phospholipids was determined by first labeling the cells with [^14^C]acetate in the presence of glycerol. The [^14^C]acetate and glycerol were then removed from the culture and the distribution of lipid classes examined after 30 min of glycerol deprivation by 2-dimensional thin-layer chromatography (Figure 2). A clear finding from these compositional analyses was that the metabolism of phosphatidylglycerol (PtdGro) continued in the absence of new synthesis (Table 1). Two polar phospholipids were detected in glycerol-depleted cells that were not detected in the glycerol-supplemented cells. These two phospholipids corresponded to the migration positions of phosphatidic acid (PtdOH) and CDP-diacylglycerol (CDP-DAG) (Figure 2B). These identifications were confirmed by the detection of increased amounts of PtdOH and CDP-DAG by mass spectrometry profiling of the phospholipid classes (Figure 3). These phospholipids would arise from the DAG formed from the transfer of the PdtGro to lipoteichoic acids (LTA). However, due to the lack of glycerol-PO_4_, PtdGro cannot be resynthesized from DAG due to the requirement of PtdGro synthase for glycerol-PO_4_ leading to the accumulation of the PtdOH and CDP-DAG intermediates. The DAG may also be converted to diglucosyl-diacylglycerol (Glc_2_DAG); however, Glc_2_DAG levels did not increase. PtdGro was also the precursor to Lys-PtdGro, and the level of Lys-PtdGro did not increase following glycerol removal indicating that the conversion of PtdGro to Lys-PtdGro was coupled to new PtdGro synthesis. A striking change was the increase in cardiolipin content from the low levels characteristic of logarithmically growing cells to 12.5% of the total phospholipid. These compositional data illustrated that after depletion of the glycerol-PO_4_ pool, PtdGro metabolism to LTA and cardiolipin continued leading to the depletion of PtdGro, and the accumulation of cardiolipin and biosynthetic intermediates due to the block at the PtdGro synthase step resulting from the absence of glycerol-PO_4_.

**Table 1:** Membrane phospholipid metabolism following glycerol deprivation

**Spot number**	**Membrane lipid**	**% total **^**14**^**C-label**	
		**W/ Glycerol**	**W/o Glycerol**
1	Phosphatidic acid	< 1	15.1
2	CDP-diacylglycerol	< 1	6.2
3	Lysyl-phosphatidylglycerol	23.2	18.4
4	Phosphatidylglycerol	55.0	28.4
5	Diglucosyldiacylglycerol	21.9	19.3
6	Cardiolipin	< 1	12.5

### Coupling of fatty acid and phospholipid synthesis

**Figure 4:**
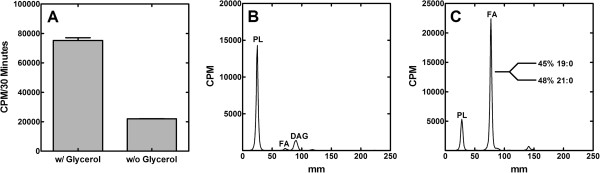
**Synthesis of lipid classes from [**^**14**^**C]acetate after blocking phospholipid synthesis at the PlsY step. (A)** Strain PDJ28 (Δ*gpsA*) was grown to an OD_600_ of 0.5, the culture was harvested, washed and split into media either with or without the glycerol supplement. The cells were then labeled with [^14^C]acetate for 30 min, the lipids were extracted and the total amount of label incorporated into cellular lipids was determined. The extracted lipids were analyzed by thin-layer chromatography on Silica Gel G layers developed with chloroform:methanol:acetic acid (98/2/1, v/v/v). The distribution of radioactivity was determined using a Bioscan Imaging detector for the cultures containing the glycerol supplement **(B)** and the glycerol-deprived cultures **(C)**. The composition of the free fatty acids that accumulated in the glycerol starved cultures was determined by preparative thin-layer chromatography to isolate the fatty acids, followed by the preparation of methyl esters and quantitative analysis by gas–liquid chromatography as described in Methods. The weight percent of the two major fatty acids detected is shown in the figure. All other fatty acids were present at less than 1% of the total.

The fate of newly synthesized fatty acids produced following withdrawal of the glycerol supplement were determined by labeling with [^14^C]acetate. The first observation was that the rate of acetate incorporation was significantly reduced, but not eliminated, in glycerol-deprived cells (Figure 4A). There was some residual synthesis of PtdGro, but the most pronounced effect was the accumulation of non-esterified fatty acids in the neutral lipid fraction (Figure 4B & 4C). Thus, the fatty acids synthesized in glycerol deprived cells were not incorporated into phospholipid, but rather accumulated as fatty acids. These fatty acids were identified by gas chromatography following their isolation by preparative thin-layer chromatography from glycerol-depleted cells. The free fatty acid pool consisted of longer chain 19:0 (45%) and 21:0 (48%) fatty acids (Figure 4C, *inset*), which were not normally abundant in *S. aureus* phospholipids. These data showed that fatty acid synthesis continued at a diminished rate in glycerol-deprived cells resulting in the accumulation of abnormally long chain length (19:0 + 21:0) fatty acids as opposed to the 15:0 + 17:0 fatty acids found in the phospholipids of normally growing cells [14]. The longer-chain fatty acids arose from the continued action of the FabF elongation enzyme in the absence of the utilization of the acyl-ACP by the PlsX/PlsY pathway.

**Figure 5:**
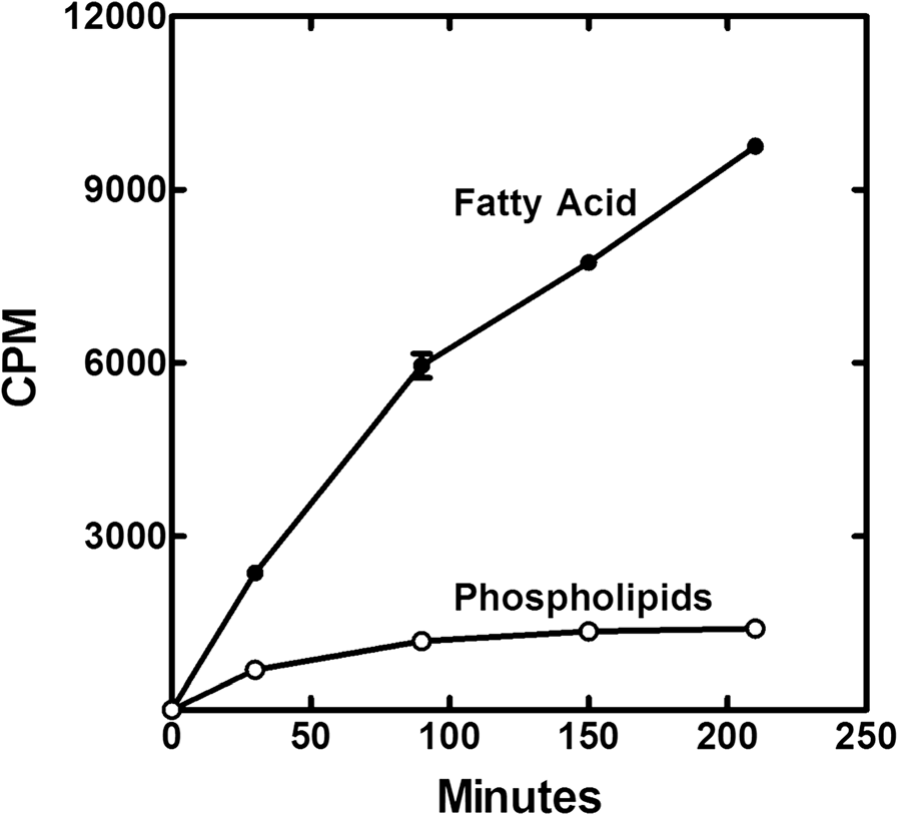
**Time course for the incorporation of [**^**14**^**C]acetate into the lipids of strain PDJ28.** Strain PDJ28 was grown to an OD_600_ of 0.5, the cells were harvested, washed and resuspended in media without glycerol. [^14^C]acetate was added to the culture 30 min after the cells were resuspended in the new growth medium, samples were removed at the indicted times, the lipids were extracted, and the distribution of label between the phospholipid and fatty acid pools were determined by thin-layer chromatography.

Next, the time course for the continued synthesis of lipids following glycerol withdrawal was determined (Figure 5). New phospholipid synthesis was noted at the first time point following glycerol deprivation and was attributed to the utilization of intracellular glycerol-PO_4_ that remained in the cells following the washing procedure. After this initial phase, phospholipid synthesis ceased. In contrast, total lipid synthesis continued, which was entirely due to the accumulation of intracellular free fatty acids. The media was extracted and analyzed, and no extracellular labeled fatty acids were detected. The accumulation of fatty acid was not a linear function of time, but rather became progressively slower. These data indicated that fatty acid and phospholipid synthesis were coupled at the PlsY step, however, the continued synthesis of free fatty acids showed that there was a biochemical pathway to bypass the regulatory steps and accumulate an intermediate that is usually not detected. The fatty acids could come from the hydrolysis of acyl-ACP, but this seems unlikely in light of the observation that fatty acids did not accumulate in a strain depleted of PlsX [23] where acyl-ACP, but not acyl-PO_4_, would be formed. Thus, it was likely that long-chain fatty acids accumulated due to the hydrolysis of the unstable acyl-PO_4_ formed from acyl-ACP by PlsX when the PlsY step was blocked by glycerol removal.

### Intracellular intermediate pools following glycerol deprivation

**Figure 6:**
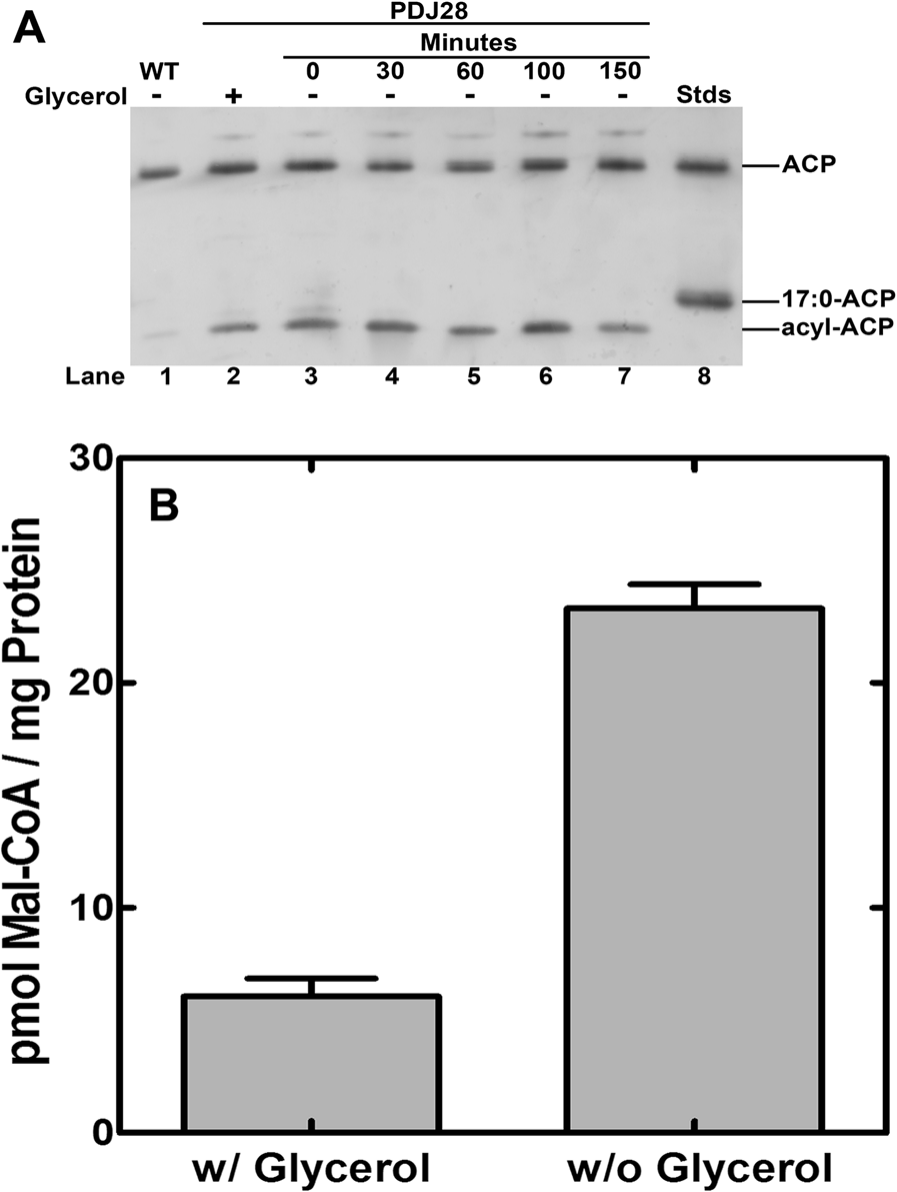
**Alteration in intracellular acyl-ACP and malonyl-CoA following the inactivation of PlsY. (A)** Cultures of strain PDJ28 (Δ*gpsA*) were grown to an OD_600_ of 0.5, samples were collected, and then the cells were washed to remove the glycerol supplement and the composition of the ACP pool determined by gel electrophoresis of the cell extracts followed by immunoblotting with anti-ACP antibody as described in Methods. **(B)** Cultures of strain PDJ28 were grown to an OD_600_ of 0.5, the culture was harvested, washed to removed glycerol and resuspended in media either with or without glycerol supplement. After 30 min, triplicate cell cultures were harvested, extracted and malonyl-CoA quantified by mass spectrometry as described in Methods.

The decrease in the overall rate of fatty acid synthesis suggested a feedback regulation mechanism that may be similar to that in *E. coli* where acyl-ACPs are key negative regulators of FASII [4]. We examined the intracellular concentrations of acyl-ACP in strain PDJ28 (Δ*gpsA*) as a function of time following glycerol withdrawal. Interestingly, we consistently observed that there was more acyl-ACP in strain PDJ28 supplemented with glycerol compared to its wild-type counterpart suggesting that PlsY activity was somewhat compromised by GpsA inactivation even in the presence of the media supplement (Figure 6A). Within 30 min of glycerol removal, the acyl-ACP pool reached 50% of the total ACP and remained constant for the remainder of the time course. The gel electrophoresis system separates acyl-ACP based the nature of the acyl chain, and the fact that the acyl-ACP in the glycerol-starved cultures migrated faster than the 17:0-ACP standard indicated that these acyl-ACP chains were longer than 17 carbons. This conclusion was consistent with the finding that 19:0 and 21:0 fatty acids accumulated in the glycerol-deprived cells (Figure 4C), and these fatty acids would be derived from the acyl-ACP end-products of *de novo* fatty acid synthesis. These data showed that acyl-ACP did accumulate in the absence of PlsY function, but that not all the ACP was converted to acyl-ACP. Rather a steady state level of acyl-ACP was attained that remained constant for hours. Also, we did not observe any acyl-ACP pathway intermediates, only the pathway end-products. This is in contrast to the effect of an enoyl-ACP reductase inhibitor, which results in almost all of the free ACP being converted to short-chain acyl-ACP [14]. These data indicated the presence of a regulatory mechanism that sensed the long-chain acyl-ACP and inhibited initiation of new acyl chains.

The lack of acyl-ACP intermediate detected in the glycerol-deprived cells suggested that there was sufficient malonyl-CoA present to complete an acyl chain once it was initiated. This question was explored by measuring the intracellular levels of malonyl-CoA in the presence and absence of glycerol by mass spectrometry (Figure 6B). These data showed that malonyl-CoA levels increased following glycerol withdrawal. This observation was consistent with the inhibition of fatty acid synthesis, but at the same time illustrated that there was sufficient malonyl-CoA present to complete the synthesis of any initiated chain in the glycerol-deprived cells. However, the levels of malonyl-CoA remained a minor component of the CoA pool. Acetyl-CoA, the substrate for acetyl-CoA carboxylase, was the most abundant CoA thioester in *S. aureus*, as it is in *E. coli*[31]. Malonyl-CoA was 0.8% of the acetyl-CoA pool in cells grown in glycerol and only rose to 3.7% of the acetyl-CoA in the cells deprived of glycerol. These data showed that acetyl-CoA carboxylase activity was also regulated in the absence of phospholipid synthesis because the cells retained a high concentration of acetyl-CoA substrate that was not consumed in the glycerol-deprived cells. The higher levels of malonyl-CoA may also have increased expression of genes controlled by FapR [16,17], although the pathway would remain blocked do the absence of glycerol-PO_4_.

## Discussion

This study reveals that the synthesis of new membrane PtdGro in *S. aureus* is not tightly coupled to its utilization by other pathways leading to a significant alteration in membrane homeostasis when phospholipid synthesis halts. Removal of the glycerol supplement from strain PDJ28 (Δ*gpsA*) results in the cessation of phospholipid synthesis, but the metabolism of PtdGro continues. One major pathway for the metabolism of PtdGro in *S. aureus* is the transfer of the *sn*-1-glycerol-PO_4_ headgroup of PtdGro to the growing LTA polymer by LtaS [32]. The DAG formed from PtdGro utilization in this pathway has two metabolic fates: 1) DAG is converted to PtdOH by DkgB [33] and recycled back toward PtdGro via CDP-DAG, or 2) DAG is converted to GlcDAG and Glc_2_DAG by YpfP [34], which serves as the scaffold for glycerol-PO_4_ polymerization in LTA synthesis. In the absence of a glycerol-PO_4_ supplement, the PtdGro in the Δ*gpsA* cells cannot be remade due to the requirement of PtdGro synthase for glycerol-PO_4_ resulting in the accumulation of PtdOH and CDP-DAG intermediates. Interestingly, the levels of neither Glc_2_DAG nor Lys-PtdGro, via MprF [35], increased in the glycerol-depleted cells suggesting that the synthesis of these two membrane lipids is linked to the synthesis of new PtdGro. A striking result was the upregulation of cardiolipin synthesis in the glycerol deprived cells. *S. aureus* possesses two cardiolipin synthase genes [36-38]. The accumulation of cardiolipin in stationary phase is attributed to Cls2, whereas cardiolipin synthesis in response to physiological stress depends on Cls1. The Cls1 stress response was rapid and does not require new protein synthesis [38]. Which of these Cls enzymes is responsible for the activation of cardiolipin synthesis in the absence of glycerol-PO_4_ remains to be determined. However, the conversion of PtdGro to cardiolipin appears to be a logical stress response to glycerol deficiency because the net effect is the release of intracellular glycerol that could be used to support PtdGro biosynthesis.

The data also suggest that the coupling of fatty acid synthesis and phospholipid has features that are similar to those observed in *E. coli*. The removal of the glycerol supplement results in diminished fatty acid synthesis that correlates with the accumulation of acyl-ACP. These accumulated acyl-ACPs are long-chain acyl-ACP end-products, and there is no evidence for the accumulation of acyl-ACP pathway intermediates. The fact that acyl-ACP does not rise to consume the entire ACP pool points to the regulation occurring at the initiation of fatty acid synthesis at the FabH step. This conclusion is consistent with the increased levels of malonyl-CoA, which indicate that the supply of malonyl groups is sufficient to complete the synthesis of an initiated acyl chain. However, malonyl-CoA levels only rose to 3.7% of the acetyl-CoA pool in the glycerol-deprived cells pointing to a biochemical regulatory mechanism that constrains the activity of acetyl-CoA carboxylase. FabH and acetyl-CoA carboxylase are key regulatory points in *E. coli* where acyl-ACP is thought to be the biochemical regulator of these two enzymes [11,12]. Our in vivo data are consistent with acyl-ACP targeting the same two proteins in *S. aureus* as in *E. coli*, but biochemical experiments will be required to verify this idea and define the mechanism of regulation.

## Conclusions

PtdGro biosynthesis is not coupled to its utilization leading to the accumulation of pathway intermediates. The synthesis of cardiolipin significantly increased revealing a stress response to liberate glycerol-PO_4_ for PtdGro synthesis. Acyl-ACP accumulation correlated with a decrease in fatty acid synthesis. However, the regulation of fatty acid synthesis was not stringent enough to prevent the accumulation of intracellular fatty acids.

## Abbreviations

glycerol-PO4: *sn*-glycerol-3-phosphate; PtdOH: Phosphatidic acid; Glc2DAG: Glucosyl-diacylglycerol; PtdGro: Phosphatidylglycerol; DAG: Diacylglycerol; ACP: Acyl carrier protein; Lys-PtdGro: Lysyl-phosphatidylglycerol; acyl-PO4: Acyl-phosphate; LTA: Lipoteichoic acid

## Competing interests

The authors declare that they have no competing interests.

## Authors' contributions

JP, JY and CR designed the study, JP, JY, MF and PJ conducted the experiments. All authors analyzed the data, and CR prepared the first draft of the article. All authors read and approved the final manuscript.
